# The expanding scope of amyloid signalling

**DOI:** 10.1080/19336896.2021.1874791

**Published:** 2021-02-12

**Authors:** Asen Daskalov, Sven J. Saupe

**Affiliations:** Institut de Biochimie et de Génétique Cellulaire (CNRS UMR 5095, Université de Bordeaux), France

**Keywords:** NLR, prion, amyloid, regulated cell death, Het-s, RHIM

## Abstract

Formation of higher-order supramolecular complexes has emerged as a common principle underlying activity of a number of immune and regulated cell-death signalling pathways in animals, plants and fungi. Some of these signalosomes employ functional amyloid motifs in their assembly process. The description of such systems in fungi finds its origin in earlier studies on a fungal prion termed [Het-s], originally identified as a non-Mendelian cytoplasmic infectious element. Janine Beisson has been a key contributor to such early studies. Recent work on this and related systems offers a more integrated view framing this prion in a broader picture including related signalling systems described in animals. We propose here an auto-commentary centred on three recent studies on amyloid signalling in microbes. Collectively, these studies increase our understanding of fold conservation in functional amyloids and the structural basis of seeding, highlight the relation of fungal amyloid motifs to mammalian RHIM (RIP homotypic interaction motif) and expand the concept of Nod-like receptor-based amyloid signalosomes to the prokaryote reign.

## Introduction

On February 24^th^ of 1958, Janine Beisson presented a paper at the *Académie des Sciences* reporting that the transformation of *Podospora anserina s^S^* strains to the *s* phenotype (now written respectively [Het-s*] and [Het-s]) is induced by a single cytoplasmic contact and that this transformation invades the mycelium at a rate of up to 7 cm per day (that is about an order on magnitude faster than the radial growth rate of the mycelium). In this paper, she proposed that this invasive [Het-s*] to [Het-s] transformation results ‘from multiplication in the [Het-s*] strains of particles brought in by the [Het-s] strain’ (‘*résulte de la multiplication dans la souche* s^s^ *de particules apportées par la souche* s’) [1]. It is now known that these particles are prions [2]. This study was based on micromanipulation techniques allowing for controlled anastomoses of individual fungal filaments and their re-isolation. Beisson would use these micromanipulation and microsurgery techniques anew in Tracy Sonneborn’s group in Bloomington in the context of their now classic common work on cortical inheritance in Paramecia [3]. Janine Beisson who sadly passed away last august once explained that her initial intent as a young student was to study the ‘transforming principle’ under the supervision of Harriett Ephrussi-Taylor who had been a co-worker of Avery. Instead, she was re-directed by Boris Ephrussi to Georges Rizet. Rizet a founding father of French genetics had introduced *Podospora* as a genetic model organism. He described in 1950, the peculiar genetics and cytology of s/S incompatibility as ‘*un phénomène tout à fait origin*al’, a thoroughly original phenomenon [4]. It is thus delightful enough that this initial impetus to pursue the ‘transforming principle’ led Janine Beisson to develop a body of work completing the DNA heredity paradigm, in the study of epigenetics of structural inheritance in both fungi and protists. The seminal studies by Rizet and Beisson in the 1950s and 1960s lay the groundwork for ongoing developments in the characterization in *Podospora* and beyond, of genetic systems related to ‘petit *s*’ *aka* [Het-s]. After a few years of practising experimental biology, one is likely to have faced at least once – when exposed to biological unorthodoxy – the choice between engaging the risky path of additional experimentation and autoclaving the whole business. In retrospect, one can only marvel at the insight and dedication of these scientists who simply would not let the *‘petit s’* genetic oddity rest unquestioned and who thus established the solid theoretical and experimental foundations, on which recent studies of such systems are based on. We will here very rapidly describe the biology of the [Het-s] prion and the path leading from the [Het-s] prion to the characterization of amyloid signalling in fungi. We then discuss three recent papers on [Het-s]-related systems that collectively expand the evolutionary span of amyloid signalling and attempt to integrate these systems into the broader picture of regulated cell death in animal, plant and prokaryotic organisms.

## [Het-s]/HET-S

Strains of the fungus *P. anserina* exist as two genotypes termed *het-s* and *het-S* and the strains of these genotypes are incompatible, meaning that cell fusions between them result in regulated cell death [5]. This occurs however only when the *het-*s-encoded protein is in its [Het-s] prion form. When in the soluble state-designated [Het-s*], the protein is inactive in incompatibility. [Het-s*] strains eventually all acquire the [Het-s]-prion state and as mentioned above, cytoplasmic contact between a prion-infected [Het-s*] strain induces rapid and invasive transformation of the [Het-s*] to [Het-s] phenotype. The HET-s protein displays a C-terminal prion-forming domain (PFD or HET-s (218–289)) comprising two 21 amino acid long pseudo-repeats (R1 and R2) adopting a specific β-solenoid amyloid fold, in which each repeat occupies one rung of β-strands [6,7]. Incompatibility is triggered when [Het-s] interacts with HET-S (‘large S’, which is an allelic variant of the same protein differing from HET-s by just a few residues). Incompatibility (*i.e*. cell death) occurs because the PFD region induces β-solenoid folding of the corresponding region in HET-S, leading to a conformation change in the N-terminal globular domain of HET-S (termed HeLo), which turns it into a pore-forming toxin by exposing a hydrophobic N-terminal α-helix [8–10]. In HET-s in contrast, amyloid conversion of the PFD does not lead to toxicity. This is because amino acid changes in the N-terminal α-helix region abolish activity of the HeLo domain. As a result, HET-s is a prion while HET-S is not. In a nutshell, in this incompatibility system, the [Het-s] prion acts as an activation switch of HET-S pore-forming activity. Remarkably, the HeLo domain shows homology to other pore-forming domains involved in regulated cell death, both in plants and animals, the four helix bundle domain of the mixed lineage kinase domain-like protein (MLKL) involved in necroptosis execution and coiled coil-related domains found in Nod-like receptors and other plant immune response proteins [11–14].

## NWD2/HET-S

**Figure 1.**
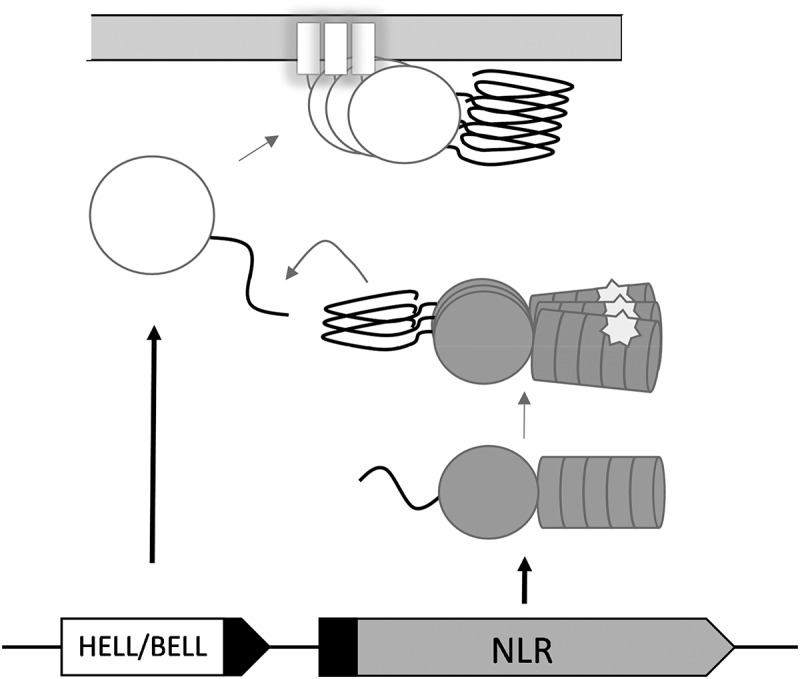
A generic scheme of NLR amyloid signalling

The allorecognition role of the *het-s* and *het-S* alleles appears however to be a derived function [15]. A distinct, evolutionarily more ancient mode of activation of HET-S is dependent on NWD2, the protein encoded by the gene immediately adjacent to *het-S* in the genome. NWD2 encodes a Nod-like receptor (NLR) with a nucleotide-binding and oligomerization domain of the NACHT type and C-terminal WD-repeats (Figure 1). NLRs constitute a diverse family of immune receptors involved in host defence both in plants and animals [16]. NLR homologs also control regulated cell death in the context of non-self recognition in fungi [17–19]. NLRs form supramolecular signalosomes (also designated supramolecular organizing centres or SMOCs [20]) when recognizing specific non-self cues or danger signals. The N-terminus of NWD2 displays a short region termed R0, homologous to the elementary repeats of the HET-S PFD [21]. This observation suggested an amyloid signalling process by which specific NLRs would engage cognate downstream cell-death execution proteins by heterotypic amyloid templating. It was found that indeed chimeric variants of NWD2 are able to induce HET-s prionisation in a ligand-dependant manner [22]. The interaction between the NWD2 R0 and the HET-s PFD modules can be recapitulated in mammalian cells. These two modules can substitute for the Pyrin domains in the pathway leading to ASC (apoptosis-associated speck-like protein containing a CARD domain) activation by NLRP3 (nucleotide-binding domain leucine-rich repeat and pyrin-containing receptor 3) [23], stressing the analogy between amyloid signalling and prion-like activation in mammalian NLR-signalosomes. While the exact function of HET-S/NWD2 is currently unclear (in particular the cognate ligand of NWD2 is not known), it is proposed that this gene pair functions in fungal immunity to induce cell death in response to parasites or pathogens. While many open questions remain regarding the biology of the NWD2/HET-S pathway, it can be considered established that fungi possess amyloid NLR-signalosomes [24]. The NWD2/HET-S system thus represents, after the mammalian necrosome (assembled through the interaction of the RHIM amyloid motifs), a second example of an amyloid signalosome [25].

This simplified sketch summarizes key features common to the *het-s/nwd2* system of *Podospora anserina* and to analogous signalosome pathways, described or predicted in other fungal and bacterial species. The signalosome components include an NLR (Nod-like receptor) (for instance NWD2) and a cell-death execution protein (for instance HET-S). The NLR and execution proteins are encoded by adjacent (or even overlapping) genes. The N-terminal part of the NLR and the C-terminal part of the execution protein share a homologous amyloid-forming region (symbolized as a black curved segment). The cell-death execution domain can be of the HeLo or HELL (HeLo-like) type in fungi or BELL type in bacteria (bacterial domain analogous to HELL). The NLR can display a range of architectures such as NACHT/WD, NACHT/ANK or NB-ARC/TPR. Upon binding of a ligand (symbolized as star-shape) in the C-terminal repeat domain, the NLR oligomerizes. This oligomerization induces cooperative folding of the N-terminal motifs into an amyloid fold. This fold is used as template to induce amyloid formation in the homologous region of the execution protein. Amyloid folding triggers activation of the pore-forming activity of the execution protein, which is dependent on the formation of a membrane-targeting α-helix and leads to induction of cell death. Many steps in this pathway remain hypothetical in some or all of the systems described in the text.

## More of the same

Bioinformatic surveys suggest that a number of genetic systems analogous to *Podospora anserina nwd2/het-S* exist in fungal genomes; gene clusters comprising a gene encoding an NLR and one or several genes encoding predicted cell death execution proteins [21] (Figure 1). These systems can be classified based on the sequence of the predicted amyloid motif, shared between the NLR and the execution proteins and have been grouped so far into three superfamilies termed HRAM (for HET-s related amyloid motifs), PP (pseudo-palindrome) and sigma [21,26]. Although the evidence is yet indirect, the sigma motif is thought to be responsible for infectious propagation of a cytoplasmic element termed σ, identified in the fungus *Nectria haematococca* [21,27]. The PP-motif in turn was functionally characterized as amyloid signalling motif as part of a study – by heterologous expression in *P. anserina –* of a three-gene cluster from the fungus *Chaetomium globosum* [12]. The PP-motif stands out among fungal amyloid signalling motifs because it shows homology to the RHIM motif involved in amyloid necrosome assembly in mammals [12,25,28]. As we shall see, recent studies have confirmed these bioinformatic predictions and support the existence of other amyloid signalosomes in fungi and also in filamentous bacteria.

## HELLF is like HET- Syet different

Accumulation of sequenced fungal genomes allowed for the identification of hundreds of HeLo domain-containing proteins, homologous to HET-S/HET-s. The *in silico* analyses resulted in the discovery of 155 homologs carrying PFDs constituted of two pseudo-repeats (R1 and R2) – an organization similar to the HET-s PFD. The pseudo-repeats (of about 21 amino acids of length) of these 155 PFDs shared sequence homology with one strongly conserved glycine residue, forming a functionally important β-arch for the [Het-s] prion, and a general pattern of hydrophobic-polar residues [26]. The putative PFD sequences were termed HET-s-related amyloid motifs (HRAMs) and grouped into five distinct families. Although the HRAMs are defined by a small number of strongly conserved positions – carrying HRAM-specific residues – it has been hypothesized that the motifs adopt a common amyloid fold, structurally related to the HET-s β-solenoid.

This hypothesis has been recently explored with the functional and structural characterization of a protein named HELLF, endogenous to *Podospora anserina* [29]. The *hellf* gene is adjacent to a gene encoding an NLR protein, *fnt1*. HELLF is a distant HET-S/s homolog constituted of 277 amino acids forming an N-terminal HeLo-like (HELL) domain and a C-terminal PFD. The pseudo-repeats comprising the PFD of HELLF belong to the HRAM5 family, which is the most diverged HRAM family from HET-s (HRAM1 family, respectively). The PFD of HELLF (HELLF(209–277)) propagated *in vivo* as a prion termed [Φ], representing a cell-death trigger for the full-length HELLF protein. The sequence identity between the pseudo-repeats of the two distant homologs was found to be as low as 17%, nevertheless it was found that HELLF(209–277) formed amyloid fibres with a β-solenoid fold virtually identical to the backbone structure of HET-s. This discovery establishes that specific amyloids folds can be maintained well into the twilight zone of sequence identity and that (as established for globular and membrane proteins) very little sequence conservation is required to maintain structural relatedness. In spite of the very strong similarity of the amyloid backbones of the β-solenoids formed by HET-s and HELLF, no cross-seeding was observed between [Φ] and [Het-s] *in vivo*. These findings demonstrate that amyloid backbone similarity does not determine alone the cross-seeding ability of prion amyloids and suggested that key side-chain residues can impact the cross-seeding between amyloidogenic sequences. The latter statement finds additional support in the observation that a designed HELLF-derived variant (termed HEC), produced by substitutions of HRAM5-specific residues in HELLF with HRAM1-specific residues from HET-s, breaches the cross-seeding barrier *in vivo* with [Het-s]. Structural examination of HEC showed that the chimeric PFD adopts a HELLF/HET-s-like amyloid fold *in vitro*, highlighting the importance of the side-chains of the HRAM-specific residues for the breach of the cross-seeding barrier between HELLF and HET-s. The designed synthetic prion HEC shows some level of structural promiscuity both *in viv*o and *in vitro* and this type of plasticity (not observed in natural HET-s–related amyloids) might be critical for the breaching of the seeding barriers. The characterization of HELLF shows that the HRAM families have diversified into signalling domains with distinct signalling specificities integrated into independent cell death pathways. The establishment of the HRAMs as a structurally unified superfamily provides a powerful experimental set-up for future investigations, because determinants specifying the amyloid signalling (ability to seed and cross-seed) must be reflected in the catalogues of natural variation found for these domains, which share a common β-solenoid fold.

## HELLP and the cross-interaction with mammalian RHIM

In addition to HELLF and HET-S, HELLP constitutes a third amyloid-activated cell-death execution protein of *Podospora anserina*. The *hellp* gene occurs in a two-gene cluster immediately adjacent to a gene encoding an NLR termed PNT1 displaying an N-terminal PP-motif, a central nucleotide-binding and oligomerisation domain of the NB-ARC type and a C-terminal TPR domain (tetratricopeptide repeats) [30]. HELLP functions in all respects analogously to HET-S and HELLF (Figure 1). The PP-motif region of HELLP forms a prion termed [π] and induces toxicity of full-length HELLP. The N-terminal PP-motif region of PNT1 also forms a prion and similarly induces toxicity and membrane localization of HELLP. Importantly, there is no cross-seeding between the respective prions formed by the HET-S, HELLF and HELLP PFDs (respectively [Het-s], [ϕ] and [π]). This absence of cross-seeding would indicate that NWD2/HET-S, FNT1/HELLF and PNT1/HELLP correspond to three parallel independently regulated cell death pathways, in which each NLR induces activation of only its cognate execution protein, as opposed to branched pathways where a given NLR could also induce other cell-death execution proteins. This last situation, at least based on bioinformatic predictions, seems however to occur in other species for which NLRs displaying similar N-terminal motifs are predicted to activate several distinct cell-death execution proteins (encoded by unlinked genes) [31]. While, the similarity of the PP-motif with the RHIM motif was noted previously this new study establishes now a functional link between RHIM and PP. Indeed, RHIM motifs of the human RIP1 and RIP3 kinases were found to behave as prions when expressed in Podospora and these prions termed [Rhim] show partial cross-seeding with [π] prions. Cross-infections are however not as efficient as in the case of homotypic [π]/[π] interactions or interactions between different [π] prions of fungal origin. These observations (in agreement with the fact that the sequence signatures of the PP and RHIM-motif differ) suggest that while there exists some level of structural relation between RHIM and PP-amyloids there must also exist marked differences lowering the cross-seeding ability. Nevertheless, the partial cross-seeding between RHIM and PP strengthens the hypothesis that these two motifs are evolutionarily related and that this type of motif has a long evolutionary history as amyloid signalling motif, involved in regulated cell death. The alternate explanation for this sequence similarity would be convergent evolution. But this hypothesis is challenged by the sequence diversity of amyloid motifs as found in fungi and bacteria (as developed below) which indicates that there are multiple evolutionary solutions to the problem of generating a short prion amyloid signalling motif (rather than a unique motif onto which different evolutionary paths would necessarily converge).

## Amyloid signalling, also in filamentous bacteria

It is important to note that the fungal amyloid NLR-signalosomes (and NLRs in general in fact) appear as far as it can be estimated from current genomic coverage limited to filamentous fungi [17,32]. Genomes of unicellular (ascomycete and basidiomycete) yeasts species do not display genes encoding amyloid signalling motifs or NLRs as identified in filamentous fungi. This is consistent with the proposed role of these NLRs in regulated cell death in the context of host-defence. While it is clear that unicellular species may also display regulated cell death pathway, immune cell death makes immediate sense in the context of multicellularity. In this context, it is of note that some bacterial proteins received a PROSITE annotation with RHIM motifs. These annotations due to the small size of the motif signature could represent false positives or *bona fide* hits oras sequences analogous to animal or viral RHIM motifs. This observation primed a bioinformatic quest for related sequences in prokaryotic genomes [33]. Specifically, it was noted that the RHIM-related motif occurs in a genomic setting reminiscent of the gene organization of fungal amyloid signalosomes, that is in gene clusters associating an NLR-encoding gene and a second gene, encoding a potential effector protein (Figure 1). Therefore, tentative amyloid signalosomes were identified based on these criteria of resemblance in overall architecture to fungal amyloid signalosomes. Amyloid motifs were clustered according to sequence similarity into 10 families designated BASS (for bacterial amyloid signalling sequences). These BASS motifs occur in proteins encoded by adjacent or overlapping genes at the N-terminus of NLRs and at the C-terminus of a protein domain termed BELL (for bacterial domain analogous to HELL). This small domain predicted to form an α-helical bundle with a hydrophobic transmembrane helix shares some sequence features with the fungal HeLo and HeLo-related domains and homologous domains involved in the control of regulated cell death in animals and plants. However there is no direct detectable sequence homology between the BELL and HeLo domains, therefore functional and structural characterization of these domains are required to precisedefine its relation to HeLo. It is currently hypothesized that BELL functions analogously as a membrane-targeting cell death-inducing domain. Interestingly as described in fungi where amyloid signalling motifs occur most frequently but not exclusively in HeLo-related domains (HeLo, HELL and sesA), the BASS-motifs do not occur only in association with BELL domains but also less frequently in association with other domains that have been implicated in host defence such as CHAT protease domains (Caspase HetF Associated with Tprs) or bacterial TIR domains (Toll/interleukin-1 receptor). The classification as 10 families is likely to represent a conservative estimate as a cut-off for the number of occurrences has been applied in the motif identification process. A lower cut-off or future expansion in genome coverage might increase the number of BASS families. Based on the current estimates however, there appears to be a greater diversity of amyloid signalling motifs in bacteria than in fungi, which in turn display more motif families than metazoans. This distribution is not surprising considering the differences in global phylogenetic diversity in these lineages and should probably not be taken as an indication that this form of signalling has experienced greater evolutionary success in multicellular bacteria than in fungi or metazoans.

The present suggestion of the existence of fungal-like amyloid NLR signalosomes points at a more general aspect of NLR function and evolution in prokaryotes and fungi. There is experimental support for the role of NLRs in fungi in the control of regulated cell death and host defence (at least in the context of allorecognition) [17]. If these putative bacterial amyloid signalosomes, but also other proteins with NLR architectures (which are also overrepresented in multicellular bacteria), function in host defence processes then a re-evaluation of the evolutionary trajectory of NLRs in the control of immunity would be required. Instead of explaining the common occurrence of NLRs in plant and vertebrate immunity as a result of convergent evolution [34], a scenario of long-term conservation of this architecture for the regulation of biotic interactions across the tree-of-life would have to be favoured [17].

## Outlook and open questions

We discuss here recent findings concerning the molecular mechanisms of NLR-associated amyloid signal transduction [29] and the evolutionary history of this class of functional amyloids [30,33]. We reflect on the origin of a signal-transducing paradigm, where signal transmission is achieved through ‘structural templating’ (or replication of a protein fold), from the early observation of non-Mendelian inheritance phenomena in *P. anserina* [1], through the discovery and characterization of the [Het-s] prion [35], to the very recent identification of signal-transducing amyloids in multicellular prokaryotes [33]. Parallel important work on RHIM and RHIM-like amyloid signalling motifs in mammals, viruses and flies further stresses the general importance of these mechanisms of signal transduction in immune pathways [36,37].

Functional amyloids in bacteria have been foremost described as constituents of biofilms [38]. Yet, our recent analyses uncovered that filamentous bacteria, mainly actinobacteria and cyanobacteria, carry two-gene clusters encoding for an NLR-like sensor and its downstream effector protein, which share a common signal-transducing amyloid motif. These findings suggest that amyloid NLR signalosomes occur in both eukaryotes (fungi) and prokaryotes (bacteria). While in fungi the amyloid signalling controls regulated cell death, which appears evolutionary related to mammalian necroptosis [12,22], the outcome of the signalling process in filamentous bacteria remains to be explored. However, it has been hypothesized that similar to the role it plays in fungi, NLR-dependent amyloid signalling represents or is integral to programmed cell death pathways in prokaryotes [33]. Future work will elucidate the extent of similarity in regards to the downstream responses of amyloid-dependent signalling between distant phylogenetic groups and likely provide additional support for a common and extremely ancient evolutionary origin of amyloid-controlled cell death. In this context, the RHIM-related amyloid motifs hold a central stage as a signalling amyloid domain common to mammals and their viruses [25,37], flies [36], fungi [12] and prokaryotes [33]. The structure of the necrosome – a heterotypic RHIM-based amyloid formed by the RHIM domains of the RIPK1 and RIPK3 kinases – has recently been resolved [39]. Our recent discovery that mammalian RHIM motifs can propagate as prions and cross-seed with the fungal PP-based prion amyloids and BASS3 bacterial amyloids [30] indicates a conserved signalling specificity and suggests, in the frame of the amyloid templating paradigm, that a degree of structural similarity exists between these signalling amyloids. Further structural exploration between these signalling amyloids would be of great interest considering that the short amyloidogenic domains are not found in similar protein architectures, specifically in mammalian signalling compared to fungi and prokaryotes. It is conceivable that some amyloid domains, like RHIM, had have higher evolutionary success due to their adaptability to form signalling complexes in a variety of protein architectures and are not strictly limited to the adaptor N-terminal domains of NLRs or the C-terminus of execution proteins. In particular, RHIM might be able in contrast to the other motifs to function in a central position as opposed to N or C-terminal positions. The fact that RHIM-like motifs also occur in Drosophila and viruses, in different protein architectures and in a central position, tends to support this view [28,36]. In turn, it must be realized that the bioinformatics approaches used to identify amyloid motifs in fungi and bacteria are biased to identify motifs occurring in the NLR/executor architectures. Further genome mining approaches to identify amyloidogenic motifs with redefined criteria would be welcome and could help answer three key questions about the phylogenetic distribution of the signalling amyloids. Are there signalling amyloids in fungi and bacteria, in proteins and signalling pathways not dependent on NLRs? Is the RHIM motif the only signalling amyloid in metazoans (and their viruses) and can the amyloid signalling paradigm be extended to other taxa, notably plants and Archaea? While experimental validation is required, it is of note that some of the amyloid motifs occurring in bacteria also occur in Archaea genomes and specifically in multicellular species [33]. The answers to these questions would bring more clarity on where we stand now in regards to diversity, abundance and distribution for this class of functional amyloids.

Aside from these structural and phylogenetic aspects, a major blind spot in these studies is the question of the biological role of the identified NLR signalling cascades. This is true for the microbial NLRs involving amyloid motifs but equally for the microbial NLRs in general. In particular, with the exception of a handful of NLRs involved in allorecognition in fungi, for none of these systems are the triggering cues known [17]. While the central connection point (the amyloid signalling motif *per se*) begins to be well defined, both the downstream events (how exactly do HeLo-related proteins bring about membrane damage?) and foremost the upstream activation steps (what are the ligands?) remain for the time being, at best, highly hypothetical. Then, an important knowledge gap resides in the definition of the actual structure of the NLR amyloid signalosomes, notably regarding the actual subunit stoichiometry of these complexes. In addition, what is unclear at present is whether the execution domains (such as HeLo) remain associated to the NLR moiety as a heterotypic amyloid assembly or whether the PFD regions receive the structural template information and then detach from the NLR amyloid hub. Thus, while some structural aspects of amyloid signalling in fungi have been explored in atomic detail [7,29], the global structural and biological understanding of these nanomachines is still in its very infancy. Important also is to articulate precisely, the relation of amyloid signalling to prion propagation. While several of the amyloid signalling components from fungi, bacteria and mammals (HELLP, HELLF, BASS and RHIM) could be artificially turned into prions, none of these systems, with the notable exception of [Het-s] (and σ in *Nectria*), occurs as natural prions. Only the latter two behave as infectious entities and cytoplasmic genetic elements transmitted between organisms. This is the very property that ignited in early studies the interest of pioneering scientists such as Janine Beisson.
